# Leber hereditary optic neuropathy in Slovenia: quality of life and costs from patient perspective

**DOI:** 10.1186/s13023-024-03329-0

**Published:** 2024-08-30

**Authors:** Marko Hawlina, Lea Kovač, Katarína Breciková, Jan Žigmond, Vladimír Rogalewicz, Aleš Tichopád, Martin Višňanský, Ivana Šarkanová

**Affiliations:** 1 Eye Hospital, University Medical Centre Ljubljana, Ljubljana, Slovenia; 2 CEEOR s.r.o., Prague, Czechia; 3 Department of Biomedical Technology, Faculty of Biomedical Engineering, Czech Technical University Prague, Kladno, Czechia; 4 Department of Pharmacy and Social Pharmacy, University of Veterinary Medicine and Pharmacy Košice, Košice, Slovakia; 5 Department of Public Economics, Faculty of Economics and Administration, Masaryk University Brno, Brno, Czechia

**Keywords:** LHON, Socioeconomic burden, Absenteeism, Productivity loss, Quality of life

## Abstract

**Introduction:**

Leber hereditary optic neuropathy (LHON) is the most commonly diagnosed mitochondrial disorder, resulting in colour vision abnormalities and rapid but painless deterioration of central vision. While numerous studies have assessed the impact of LHON on the quality of life (QoL) of LHON patients, the financial impact of the disease remains unexplored. This study attempts to calculate both the direct non-medical costs and the indirect costs associated with productivity losses experienced by people with LHON and their unpaid caregivers in Slovenia, in addition to assessing their QoL. Due to the rarity of the disease, the study involved a small sample size, which is important to note for interpreting the results.

**Methods:**

The analysis was conducted on nine adult participants diagnosed with LHON, representing one-third of the total number of known Slovenian patients with this condition. To thoroughly assess the economic and social impact of LHON, tailored questionnaires were designed to collect information on demographics, socioeconomic status, LHON severity, and associated non-medical and indirect costs.

**Results:**

The mean age of the study participants was 48.8 years (SD 13.3; *n* = 9). The annual productivity loss attributable to LHON, taking both absenteeism and relative presenteeism into account, was calculated to be EUR 11,608 per person affected. The mean VFQ-25 score, a measure of vision-related quality of life, for adult LHON patients was 30.4 (SD 12.9).

**Conclusion:**

The findings highlight the significant economic and social burden of LHON on patients and their families. Ensuring prompt, accurate diagnosis, access to treatment, financial support, and psychological counselling and services are critical to helping individuals cope with and mitigate the profound challenges of vision loss and living with LHON.

## Introduction

Leber’s Hereditary Optic Neuropathy (LHON) is the primary mitochondrial disease affecting retinal ganglion cells, which is characterized by colour vision defects and a gradual, painless loss of central vision. In 25–50% of cases, both eyes are affected simultaneously. This condition mainly affects young adults, with the highest incidence occurring in their twenties and thirties [1, 2]. Men are more likely to develop LHON than women, with a three to five-fold increase in risk. More than 90% of LHON cases are associated with one of three primary mitochondrial DNA mutations: m.3460G > A, m.11778G > A, and m.14484T > C. These genetic changes disrupt the function of complex I (NADH-ubiquinone oxidoreductase) in the mitochondrial respiratory chain, leading to impaired energy production and subsequent cell death. The mutations remain asymptomatic until triggered by certain unknown factors that lead to the manifestation of the disease [3–5]. Recently, autosomal recessive forms of LHON (DNAJC30 gene) have also been described and are increasingly reported especially from countries of Central and Eastern Europe [6, 7].

LHON is a rare condition, occurring in approximately 1 in 30,000 to 1 in 54,000 individuals across Europe [8–10]. However, when considering both symptomatic individuals and asymptomatic carriers with confirmed genetic mutations, the estimated frequency increases to about 1 in 8,500 [11]. In 2021, in Slovenia there were 27 genetically confirmed and symptomatic LHON cases, resulting in an estimated prevalence rate of 1 in 72,000 [12], however if all genetically yet unconfirmed affected cases in LHON families would be counted in, the prevalence is estimated to be around 1 in 50,000.

The prognosis for maintaining normal visual function in LHON patients is generally poor, as the extent of vision loss is following the progressive pattern from the first symptoms to consecutive vision loss on both eyes. Currently, there is no known therapy to either halt the onset of LHON or completely restore vision. Idebenone, a synthetic version of coenzyme Q10, is the primary treatment option approved for LHON patients [13]. Real-world practice suggests that idebenone may aid in the restoration of lost vision and preservation of existing vision quality, particularly in patients treated early (within a year of disease onset) with a regimen of 900 mg/day for a minimum of two years, aiming for optimal results [5, 14, 15]. However, it is currently under observation if chronic patients may as well benefit from idebenone therapy as reported by several authors [16, 17].

The impact of LHON extends beyond the individual and imposes a significant socio-economic burden on families due to reduced productivity and diminished quality of life (QoL). Although several studies have examined the QoL of LHON patients [18–20], a broader economic impact, including both direct non-medical and indirect costs such as productivity losses, has not been explored. This study seeks to fill that gap by evaluating the direct and indirect expenses borne by LHON patients in Slovenia, in addition to assessing their QoL.

## Materials and methods

### Landscape mapping

Prior to patient data collection, an in-depth face-to-face interview with a key opinion leader (KOL) on LHON in the Slovenia was conducted to gain a deep insight into the disease, including availability of diagnosis and treatment, patient journey, patients’ number etc.

### Participants

The study was conducted with 9 adult patients with LHON in Slovenia. The study received approval from the Medical Ethics Committee of the Ministry of Health of the Republic of Slovenia under the reference number 0120–407/2022/13. All participants provided informed consent.

### Study design

The study was performed in Slovenia. Data were collected from 9 adults with LHON. To determine the socioeconomic burden of LHON, a questionnaire was developed for adult patients, including demographic and socioeconomic data. The centre recruited patients through their treating physicians. All LHON patients in the centre’s database were offered the opportunity to participate in the study on a voluntary basis and were provided with a link to an online questionnaire. The analysis was conducted on the responses of nine patients who had consented to participate and completed an anonymous online questionnaire, representing one-third of the total number of known Slovenian patients with this condition. The analysis was conducted on the responses of 9 patients who agreed to participate and completed an anonymous online questionnaire. The following data were collected: age, education, family size, severity of LHON, non-medical direct and indirect costs of LHON. Direct non-medical costs included out-of-pocket payments for transport, education, informal care (adult patients), visual aids and home modifications. These costs were calculated on the basis of the survey results. Indirect costs (patient productivity losses) were calculated as absenteeism and presenteeism according to the World Health Organization’s Heath and Work Performance Questionnaire (HPQ) [21]. Absenteeism is defined as “absence from work due to health problems”, while presenteeism is defined as “health-related loss of productivity at paid work”.

Data on quality of life were also collected using the National Eye Institute 25-item Visual Function Questionnaire (VFQ-25, 2000 version) [22]. The VFQ-25 consists of 25 vision-related questions representing 11 vision-related constructs and an additional single-item general health assessment question. VFQ-25 subscale scores are the average of the list items in the subscale transformed to a 0-100 scale, where 100 is the best possible score on the measure and 0 is the worst. The VFQ-25 composite score is an unweighted average of responses to all list items except the general health assessment question [22].

### Data collection and analysis

Data were collected using an electronic data collection system (CLADE-IS). All data collected via CLADE-IS were checked for clarity and completeness by the data managers of CEEOR. Descriptive data analysis was primarily used for all variables of interest. Ordinary and categorical variables were analysed using frequencies. All metric data were described by mean, standard deviation, median, minimum and maximum. Linear correlation and unpaired t-test were used to assess whether the age of patients could affect the measured outcomes of quality of life. Absenteeism was scored in terms of hours lost per month, i.e. a higher score indicates a higher level of absenteeism. We calculated the monetary value of a working hour for patients who were absent due to LHON and then multiplied by the number of working hours missed to estimate the cost of absenteeism. Relative presenteeism was calculated as the ratio of actual performance to the performance of most workers in the same job. Productivity losses were assessed using the human capital approach (HCA) [21], which expresses the loss as the product of missed hours multiplied by the national average gross hourly wage (€ 13.12) in 2023 (https://www.stat.si/statweb/en/Field/Index/15/74).

The cost of informal care was calculated using the opportunity cost method, i.e. the number of hours missed from work by informal carers was multiplied by their hourly wage.

Analyses were performed using SAS 9.4 software, version TS Level 1M7 × 64_10PRO platform.

## Results

### Mapping the landscape

The table below summarises in-depth insight and perspective on the disease, including centres, number of patients, diagnosis, reimbursement system and treatment, collected from the local KOL (Table 1).

**Table 1:** Landscape mapping of LHON in Slovenia

	Slovenia
**Centre for LHON treatment**	Eye Hospital, University Medical Centre Ljubljana
**No of patients in centre’s database**	29 genetically confirmed and symptomatic
**Average time to diagnosis**	2 months from the onset of LHON
**Diagnostic markers**	Genetic confirmation/fluorescein angiography/SD-OCT/electrophysiology
**Reimbursement system of idebenone (Raxone)**	Reimbursement listed
**Treatment**	Treatment initiated within 6 months from the onset of LHON as a standard

Abbreviations: SD-OCT (Spectral Domain Optical Coherence Tomography)

### Epidemiology

Of the 9 patients with LHON included in the study, 6 (66.7%) were males and 3 (33.3%) were females. The mean age of all respondents was 48.8 years (SD 13.3, range 31–65), in males 48.2 years (SD 14.3; range 31–63) and in females 50.0 years (SD 13.7; range 38–65).

The most common level of visual impairment measured by self-report was ‘some useful peripheral vision’, followed by ‘light (or shadow) perception only’ (Table 2). 56% (*n* = 5) of patients were treated with idebenone.

**Table 2:** Demographics

	Adults
**No of individuals**	9
**Sex**,** n (%)**	
* Male*	6 (66.7%)
* Female*	3 (33.3%)
**Age**,** Years**	
* Mean (SD)*	48.8 (13.3)
* Range*	31–65
**Severity of visual impairment**,** n (%)**	
* Good peripheral vision*	1 (19%)
* Some useful peripheral vision*	6 (33.3%)
* Light perception only (or shadows only)*	2 (9.5%)
**Treatment with idebenone**,** n (%)**	5 (55.6%)

### Loss-of-productivity costs

33% of patients were receiving a pension and 33% were in full-time employment. Only 22% of patients received a disability pension (Table 3). The employed patients were not absent from work due to illness. Relative presenteeism was 53% in patients (Table 4), which means that patients had reduced performance and quality of work by about 47% due to LHON. The loss of productivity as a result of LHON due to combined relative absenteeism and relative presenteeism was estimated at € 11,608 per patient/year. Disability pensions accounted for € 8,086 per person/year and unemployment support for € 9,524 per person/year.

**Table 3:** Frequency of employment status of adult patients

Employment status	Male	Female	Total
*Disability pension*	**2 (33.3%)**	**0 (0%)**	**2 (22.2%)**
*Pension*	2 (33.3%)	1 (33.3%)	3 (33.3%)
*Full time work*	2 (33.3%)	1 (33.3%)	3 (33.3%)
*Unemployed*	0 (0%)	1 (33.3%)	1 (11.1%)

**Table 4:** Mean absenteeism and presenteeism (in %)

Adult patients	Mean	Median	Min	Max	Total costs per person/year
*Relative absenteeism*	0	0	0	0	€ 0
*Relative presenteeism*	47	25	25	90	€ 11,608
*Combination relative absenteeism and presenteeism*	47	25	25	90	€ 11,608
*Informal care (missed hours per day)*	1.0 h	1.0 h	0	5	€ 3,149

### Non-medical direct costs

Among the study participants, the majority (7 of 9 patients) required assistance with activities of daily living. Six respondents indicated that family members provided the majority of this assistance, while one patient had a full-time paid carer funded by public funds. In the case of patients with informal caregivers, one respondent reported that the caregiver’s working hours had been reduced by one hour per day, resulting in an annual cost of €3,149.More than half of the patients (55.6%) needed more than two hours for a round trip to see their specialist or ophthalmologist, with each trip costing an average of € 19.4. Expenditure on visual aids or home adaptations averaged € 117 per person per year. Rehabilitation services were used by only two patients, with an average cost of € 110 per person. In addition, four people opted for complementary treatments or support, at an average annual cost of € 276 per person.

### Quality of life

The mean VFQ-25 score for patients with LHON was 30.4 (SD 12.9; Min 15.2, Max 53.4) suggesting generally low visual functioning and quality of life. The distance activities sub-scale had the score (20.4; SD 15.1; Min 0, Max 41.7), which was the lowest among other vision targeted sub-scale scores and reflecting poor performance and substantial variability among patients, also reflected in low scores of other vision-related categories and social functioning. Ocular pain scores are highest, with a mean of 54.2, indicating relatively mild ocular discomfort. Mental health scores show a mean of 47.9, suggesting moderate mental health issues (Table 5).The potential influence of age on patients’ perceptions of quality of life was evaluated using linear correlation and unpaired t-tests. No statistically significant correlation was found.

**Table 5:** Mean score of VFQ-25 scale

Adult patients	Mean	SD	Median	Min	Max
*Total VFQ25_score*	30.4	12.9	28.9	15.2	53.4
*General health*	38.9	22.1	50.0	0.0	75.0
*General vision*	26.7	14.1	20.0	20.0	60.0
*Ocular pain*	54.2	20.7	50.0	37.5	100.0
*Near activities*	24.1	20.6	25.0	0.0	66.7
*Distance activities*	20.4	15.1	25.0	0.0	41.7
*Social functioning*	25.0	17.7	18.7	12.5	62.5
*Mental health*	47.9	21.4	37.5	25.0	81.3
*Role difficulties*	26.4	25.3	12.5	0.0	62.5
*Dependency*	43.5	23.8	41.7	25.0	100.0
*Driving*	50.0	.	50.0	50.0	50.0
*Colour vision*	34.4	29.7	25.0	0.0	75.0
*Peripheral vision*	25.0	21.6	25.0	0.0	75.0

**Discussion**

To our knowledge, this study is pioneering in that it details both the direct non-medical and indirect costs associated with LHON and is the first study of the quality of life in people with LHON in the South East Europe region. The establishment of the Eye Hospital at the University Medical Centre in Ljubljana, providing comprehensive multidisciplinary care and also training for local and international ophthalmologists and neurologists, has improved and accelerated the diagnosis of LHON. This has been instrumental in facilitating the prompt initiation of idebenone treatment that is at the moment the only registered treatment for LHON. In Slovenia, the prevalence of LHON is estimated to be 1 in 72,000, which is lower than prevalence rates reported in other European studies. However, the number refers to genetically confirmed cases, if all affected in LHON families that did not wish to be tested would be counted in, the prevalence would increase to about 1 in 50,000 which is at the low end of prevalence spectrum. In addition, a higher incidence of atypical LHON variants has been observed in the Slovenian population [12].

Several studies have shown that the financial impact of rare diseases is mainly driven by indirect and non-medical costs, which exceed those directly related to medical care [23–25]. Consistent with these findings, our research highlights that people with LHON experience significant economic burden and reduced quality of life. Specifically, those with symptoms of LHON have a reduced ability to fully participate in the workforce compared to the general population. While the participants of our study did not report absenteeism, it was presenteeism - the phenomenon of being present at work but working at a reduced capacity – that emerged as a significant contributor to lost productivity. We assessed the cost of lost productivity due to presenteeism at €11,608 per person.

Among the Slovenian patients with LHON, the average VFQ-25 score was lower at 30.4 (SD = 12.9) than that of other published study (25.1, SD 20.8) [19], indicating a pronounced reduction in overall quality of life related to vision. The range of scores, from 15.2 to 53.4, suggests a spectrum of disease expression and adaptation among patients. These findings highlight the variable impact of LHON on individuals’ daily functioning and well-being. The lowest mean score of 20.4 was found for the distant activities subscale, with low scores also with other activities related to vision indicating these are the most pronounced categories causing reduction of quality of life. In contrast, the ocular pain sub-scale presented the highest mean score of 54.2, suggesting that patients with LHON, as expected, experience less ocular discomfort compared to other symptoms of the condition.

The age at which the disease begins can affect certain aspects of quality of life. Previous research has shown that vision impairment caused by LHON has a significant impact on the psychological and social well-being of adolescents and young adults [26]. Our cohort also showed important impairment in social functioning and role difficulties (mean score 25.0 and 26.4 respectively). The self-reported mental health sub-scale scores range from 25.0 to 81.3, but we did not find any correlation between the scores and age, even when considering the severity of the condition. However, two patients with most severe impaired vision (light perception only/ shadows only) had the lowest score on the mental health scale. Interestingly, relatively high mean score of 43.5 for dependency show that LHON patients retain relatively independent life attitude.

Quality of life was significantly reduced in all participants, with scores for each sub-domain varying among patients. This indicates a highly individual perception of the disability and its impact on different areas of life and associated coping.

The study of the socio-economic impact or quality of life of patients with rare diseases is always faced with the same challenges. The small sample size reduces the statistical power, making it more challenging to detect meaningful differences or correlations. Additionally, the limited sample size increases the susceptibility of results to the influence of outliers and random variation. As even within a single rare disease, there can be a wide variability in symptoms, progression, and severity. This heterogeneity complicates the ability to generalise findings and develop standardised assessment tools. The results of this study face the same limitations and should be viewed as exploratory and indicative. Despite these challenges, conducting studies on burden of rare diseases is crucial. These studies provide valuable insights into patient needs, guide personalised care, and inform policymakers for better resource allocation. Such studies help to fill knowledge gaps, validate patient experiences, and pave the way for larger-scale research, thereby contributing to improved outcomes and awareness for the rare disease community.

### Limitations of the study

The study has several limitations, the foremost being the sample size. LHON is a rare condition with a low prevalence rate of approximately 20–30 symptomatic individuals per one million in Europe, making it inherently challenging to gather a larger sample size. However, we believe that any contribution to research on this topic is valuable, and our sample size aligns with those of similar studies. A potential area for future exploration could be a meta-analysis that synthesises existing findings, although this presents its own set of challenges.

Furthermore, our study used a self-reported survey distributed exclusively online, which introduces potential sources of bias. Although we aimed to reach all individuals in the register, only those who chose to participate were included, resulting in a convenience sample. The online format of the survey may have resulted in biased participation depending on the level of available help and ability to answer and understand rather complex questionnaires.

## Conclusion

The findings of this study clearly demonstrate the significant socioeconomic challenges faced by patients with LHON and their families as a result of this rare disease. Providing patients and their families with early, prompt, and appropriate access to diagnosis, treatment, and reimbursement options, along with psychological counselling and services ideally offered within the treatment center, could significantly assist in their adaptation and coping with the difficult realities of vision loss and living with the disease.

## Data Availability

Data from the study is outside of CHIESI’s data sharing policy and are available only upon request from the authors.

## Abbreviations

HCA: Human capital approach
HPQ: Heath and Work Performance Questionnaire
KOL: Key opinion leader
LHON: Leber hereditary optic neuropathy
QoL: Quality of life
VFQ-25: Institute 25-item Visual Function Questionnaire
SD: Standard deviation
